# The Role of Long Non-Coding RNAs in Epithelial-Mesenchymal Transition-Related Signaling Pathways in Prostate Cancer

**DOI:** 10.3389/fmolb.2022.939070

**Published:** 2022-07-18

**Authors:** Dexin Shen, Hongwei Peng, Caixia Xia, Zhao Deng, Xi Tong, Gang Wang, Kaiyu Qian

**Affiliations:** ^1^ Department of Urology, Zhongnan Hospital of Wuhan University, Wuhan, China; ^2^ Wuhan Research Center for Infectious Diseases and Cancer, Chinese Academy of Medical Sciences, Wuhan, China; ^3^ Department of Biological Repositories, Zhongnan Hospital of Wuhan University, Wuhan, China; ^4^ President’s Office, Zhongnan Hospital of Wuhan University, Wuhan, China; ^5^ Human Genetic Resource Preservation Center of Hubei Province, Wuhan, China

**Keywords:** prostate cancer (PCa), long non-coding RNA (lncRNA), epithelial-mesenchymal transition (EMT), androgen receptor (AR), wnt/β-catenin

## Abstract

Prostate cancer (PCa) is one of the most common male malignancies with frequent remote invasion and metastasis, leading to high mortality. Epithelial-mesenchymal transition (EMT) is a fundamental process in embryonic development and plays a key role in tumor proliferation, invasion and metastasis. Numerous long non-coding RNAs (lncRNAs) could regulate the occurrence and development of EMT through various complex molecular mechanisms involving multiple signaling pathways in PCa. Given the importance of EMT and lncRNAs in the progression of tumor metastasis, we recapitulate the research progress of EMT-related signaling pathways regulated by lncRNAs in PCa, including AR signaling, STAT3 signaling, Wnt/β-catenin signaling, PTEN/PI3K/AKT signaling, TGF-β/Smad and NF-κB signaling pathways. Furthermore, we summarize four modes of how lncRNAs participate in the EMT process of PCa *via* regulating relevant signaling pathways.

## Introduction

Prostate cancer (PCa) is one of the most common malignancies in males worldwide and has become a global “killer” threatening the health of elderly men. The incidence and mortality of PCa rank second and fifth in men worldwide, respectively (Sung et al., 2021). Accounting for 27% of diagnoses and 11% deaths among all the male malignancies, PCa currently had the highest incidence rate and became the second most common cause of cancer-related death in the United States (Siegel et al., 2021; Siegel et al., 2022). Although the incidence of PCa in China is lower than that in American and African countries, the incidence and mortality have increased dramatically in the past 2 decades, and the annual increase ranks first among male malignant tumors (Sun et al., 2020). PCa is a hormone-dependent disease, therefore therapies targeting the androgen receptor (AR) signaling, especially the androgen deprivation therapy (ADT), become the main clinical theory for PCa patients (Ishikawa et al., 1989; Shore et al., 2020). Even though most PCa patients present a satisfying response to ADT for a while, the disease may finally develop into castration-resistant prostate cancer (CRPC) stage, characterized by resistance to ADT and aggressive metastases in a considerable proportion of patients (Galletti et al., 2017). The 5-year survival rate for localized PCa was 99.3%, while that for metastatic PCa decreased sharply to 32.3%, demonstrating that metastasis is the leading cause of PCa-related mortality (Heidenreich et al., 2014; Siegel et al., 2020).

For tumor metastasis, EMT is an important initiating factor driving this process and plays a critical role, which confers metastatic characteristics on cancer cells by increasing mobility and invasion (Nakazawa et al., 2017). EMT is a reversible process in which relatively stable epithelial cells lose cell polarity and intercellular adhesion, and transform into spindle-shaped mesenchymal cells with migration ability (Kalluri and Weinberg, 2009). During the process of EMT, the level of multiple epithelial cell markers would decrease, such as cytokeratins, laminin and E-cadherin, which lead to the loss of cell-to-cell adhesion. In contrast, mesenchymal markers, such as vimentin, N-cadherin, *β*-catenin and Snail protein, are up-regulated, thereby allowing the cells to migrate or metastasize to different organs (Odero-Marah et al., 2018). While EMT prevents cell apoptosis and senescence, it will also cause organ fibrosis and promote tumor development and metastasis (Bhangu et al., 2012).

LncRNAs are non-coding RNAs with length greater than 200 nucleotides and hundreds of them are dysregulated in human tumors with complex regulatory mechanisms (Yu and Shan, 2016). LncRNAs have been reported to regulate the development of EMT by targeting EMT-related inducible transcription factors such as Twist, Snail, Slug and Zeb (Peinado et al., 2007). Recent studies indicated that a variety of signaling pathways regulated by lncRNAs promoted EMT and led to tumor metastasis (Montano and Bushman, 2017; Yeh et al., 2019).

The metastasis of PCa, which leads to treatment failure and death of patients, is attributed to multi-system and multi-level pathological alterations affected by multiple factors, but the molecular mechanism is not yet fully understood (Hussain et al., 2020; Subudhi et al., 2020). In this review, we aimed to recapitulate the present studies of lncRNAs in PCa and their regulation in EMT process, as well as the signaling pathways regulated by EMT-related lncRNAs in PCa.

## EMT and Tumor Metastasis

EMT occurs during different stages of embryonic development and can be classified into three distinct functional types based on their biological contexts, ranging from type I contributing to gastrulation and organ development found in normal embryological development to type III contributing to increased cancer cell invasiveness found in cancer progression and metastasis (Kalluri and Weinberg, 2009). EMT could be induced by a set of specific transcription factors including members of the Snail, ZEB and Twist families (Gheldof and Berx, 2013), which could directly regulate E-cadherin expression (Montanari et al., 2017). Supplementally, the downregulation of E-cadherin is regarded as a defining event when EMT occurred (Lo et al., 2017).

Previous studies have confirmed that the activation of EMT permitted cancer cells to acquire invasion and migration during cancer progression (Liao and Yang, 2017). A variety of molecular mechanisms regulate EMT directly or indirectly (Montanari et al., 2017; Hao et al., 2019). For example, the combination of growth factors or cytokines, such as transforming growth factor-β (TGF-β), epidermal growth factor (EGF) and insulin-like growth factor (IGF), with corresponding receptors participant in EMT *via* inducing downstream effectors (Maruyama, 2014). In addition, several signaling pathways including AR signaling pathway, PI3K/AKT pathway and Wnt/β-catenin pathway also play pivotal roles in orchestrating EMT and metastatic responses of PCa by cooperating to induce full EMT responses (Lamouille et al., 2014).

## LncRNAs and Prostate Cancer

LncRNAs are non-coding RNAs with length greater than 200 nucleotides, which are key regulatory molecules in cells and can function via various paradigms (Wilusz et al., 2009). LncRNAs could be targeted to specific DNA sequences in cis or trans, and this feature gives them the ability to regulate tumor-related target genes at different levels, including epigenetic level, transcription level and post-transcriptional level (Marchese et al., 2017; Ni et al., 2019; Braga et al., 2020; Wang et al., 2020). Some lncRNAs are specifically transcribed and participate in the transduction mediated by special signaling pathways as signal molecules. In addition, lncRNAs are capable of folding into secondary and tertiary structures or sponging miRNA to perform more complex functions (Misawa et al., 2017). In recent years, many studies have shown that lncRNAs have a significant role as regulators of key cellular processes in cancers, including PCa (Prensner and Chinnaiyan, 2011; Liu et al., 2020; Hu C -Y et al., 2021; Tan et al., 2021).

LncRNAs may act as oncogenes or tumor suppressors in PCa and participate in the invasion and metastasis of PCa (Xu et al., 2019; Lu et al., 2021). Functionally, lncRNAs possess important application potential in the occurrence and development, early diagnosis, treatment and prognosis of PCa (Ramnarine et al., 2019; Morgan et al., 2021). An influential application of lncRNA in PCa diagnosis is the detection of PCA3 (Hessels and Schalken, 2009; Morgan et al., 2021), which could regulate the expression of AR signaling pathway and critical genes involved in the carcinogenesis and development of PCa, including EMT-related genes (Ferreira et al., 2012; Lemos et al., 2019; Ghafouri-Fard et al., 2022). LncRNAs are closely correlated with Gleason score, TNM stage and PSA kinetic parameters in PCa, which are recognized as the basis for treatment decision-making and prognosis. The expression of lncRNA TINCR was measured in 160 PCa specimens, and the results revealed that low expression of TINCR was strikingly associated with advanced clinical T stage, lymph node involvement, distant metastasis and high Gleason score (Dong et al., 2018). Besides, lncRNAs are often used as crucial biomarkers in the clinic for the prediction of PCa. For example, single nucleotide polymorphisms (SNPs) in lncRNA PCAT19 was used as a predictor of PCa risk variant based on decreased and increased levels of PCAT19-short and PCAT19-long (Hua et al., 2018). LncRNAs that contribute to tumor proliferation, invasion and metastasis in PCa and may function as oncogenes or tumor suppressors are shown in Table 1.

**TABLE 1:** Different lncRNAs and their regulation in EMT of PCa.

LncRNAs	Gene ID	Expression in PCa	Regulation method	Effect on genes	Functions in PCa	Cell lines	Molecular mechanism	References
TINCR	257000	Down-regulated	Bind to protein	Tumor suppressor	Inhibit proliferation, migration and invasion	LNCaP, PC-3, DU145, 22Rv1, P69 and RWPE-1	Modulate TRIP13 mRNA and protein expressions	Dong et al. (2018)
HCG11	493812	Down-regulated	PI3K/AKT signaling pathway	Tumor suppressor	Inhibit proliferation, migration and invasion	LNCaP, PC-3, C4-2B, HEK293T and RWPE1	Inhibit PI3K/AKT signaling pathway by downregulating miR-543 expression	Wang et al. (2019)
CCAT2	101805488	Up-regulated	Bind to protein	Oncogene	Promote EMT, proliferation, migration and invasion	DU145, 22RV1 and WPMY-1	Abrogating N-cadherin, vimentin expression and intensifing the expression levels of E-cadherin	Zheng et al. (2016)
LncRNA-ATB	114004396	Up-regulated	ERK and PI3K/AKT signaling pathways	Oncogene	Stimulate EMT and inhibit growth	PC-3 and DU145	Activate ERK and PI3K/AKT signaling pathways by ZEB1 and ZNF217	Xu et al. (2016)
LINC01296	503638	Up-regulated	PI3K-Akt-mTOR signaling pathway	Oncogene	Promote proliferation, migration, and invasion	22Rv1, LNCaP and WPMY1	Regulate PI3K-Akt-mTOR signaling pathway	Wu et al. (2017)
PVT1	5820	Up-regulated	Bind to protein	Oncogene	Promote proliferation, invasion, and metastasis	PC-3, DU145, 22RV1 and WPMY	Act as a sponge for miRNA-186-5p and positively regulates Twist1	Chang et al. (2018)
PCA3	50652	Up-regulated	Modulate proteins	Oncogene	Promote growth	LNCaP	Modulate the expression of key cancer-related genes of EMT markers	Lemos et al. (2016)
PlncRNA-1	100506428	Up-regulated	TGF-β1 pathway	Oncogene	Promote growth	LNCaP, C4-2, DU145, PC-3 and RWPE-1	Regulate the growth of prostate cancer cells and EMT through the TGF-β1 pathway	Jin et al. (2017)
SNHG1	23642	Up-regulated	Through the SNHG1-hnRNPL-CDH1 axis	Oncogene	Promote EMT, proliferation and migration, accelerate xenograft tumor growth	LNCaP, 22Rv1, PC-3, DU145 and RWPE-1	Competitively interact with hnRNPL to impair the translation of protein E-cadherin, thus activating the effect of SNHG1 on the EMT pathway	Tan et al. (2021)
SNHG7	84973	Up-regulated	Through miR-324-3p/WNT2B axis	Oncogene	Promote migration and invasion	LNCaP, PC-3, Du-145 and RWPE	Promote EMT via miR-324-3p and WNT2B	Han et al. (2019)
MALAT1	378938	Up-regulated	Activating PI3K/Akt signal pathway	Oncogene	Promote proliferation, invasion, migration and inhibite apoptosis	PC-3	Inhibit EMT process and PI3K/Akt signaling pathway via downregulating MALAT1	Lu et al. (2020)
MALAT1	378938	Up-regulated	Serve as a ceRNA	Oncogene	Promote migration, invasion and EMT	DU145, PC-3, LNCaP, 22RV1 and RWPE2	Compete with CORO1C for the binding sites of miR-1-3p	Dai et al. (2019)
MNX1-AS1	645249	Up-regulated	mRNAs and proteins	Oncogene	Promote proliferation, migration, and invasion of prostate cancer	LNCaP, DU145, PC-3, C4-2 and RWPE	Promote the proliferation via regulating PCNA and PH-3	Li Z et al. (2019)
HOXA-AS2	285943	Up-regulated	Serve as a ceRNA	Oncogene	Promote proliferation, migration, invasion and influence EMT	LNCaP, DU145, PC-3 and RWPE	Serve as a competing endogenous RNA through sponging miR-509-3p to release pre-B-cell leukemia homeobox 3 expression	Xiao and Song, (2020)
PCAT7	101928099	Up-regulated	TGF-β/SMAD signaling	Oncogene	Promote bone metastasis as well as migration, invasion, and EMT	LNCaP, PC-3, 22RV1, VCaP, DU145 and RWPE-1	Activate TGF-β/SMAD signaling by upregulating TGFBR1 expression via sponging miR-324-5p	Lang et al. (2020)
ZFAS1	441951	Up-regulated	Serve as a ceRNA	Oncogene	Promote cell viability, proliferation, migration, invasion and the occurrence of EMT, inhibit apoptosis	PC-3, DU145, 22RV1, LNCAP and RWPE-1	Competitively bind to miR-135a-5p which targets the mRNA	Pan et al. (2020)
TUG1	55000	Up-regulated	Through miR-128-3p/YES1 axis	Oncogene	Promote proliferation, migration, invasion, EMT, and inhibit apoptosis	PC-3, DU145 and RWPE-1	Modulate YES1 expression by sponging miR-128-3p which interacted with TUG1 or YES1	Hao et al. (2020)
VIM-AS1	100507347	Up-regulated	Through regulating vimentin	Oncogene	Promote EMT, cell growth, proliferation, migration and invasion	LNCaP, DU145, 22RV1, PC-3 and RWPE-1	Promote the expression of vimentin and further promote EMT	Zhang Y et al. (2019)
HULC	728655	Up-regulated	Unclear	Oncogene	Promote cell growth and metastasis	LNCaP, PC-3 and DU145 and RWPE-1	Regulate the expression of N-cadherin, vimentin and E-cadherin, but the expression pattern and biological function of HULC remain largely unclear	Zheng T et al. (2018)
SNHG17	388796	Up-regulated	Serve as a ceRNA	Oncogene	Promote proliferation, invasion, migration, and EMT and inhibit apoptosis	DU145, LNCaP, VCaP, PC-3 and RWPE-1	Sponge miR-339-5p to upregulate signal transducer and activator of transcription 5A and therefore to cause transactivation of SNORA71B	Wu J et al. (2020)
KCNQ1OT1	10984	Up-regulated	Serve as a ceRNA and the Ras/ERK signaling	Oncogene	Promote viability, migration, invasion and EMT and inhibit apoptosis	DU145 and PC-3	Sponge miR-15a and release its inhibition on PD-L1 and regulate the Ras/ERK signaling	Chen et al. (2020)

## EMT-Related Signaling Pathways Regulated by lncRNAs in PCa

The tumorigenesis and development of PCa is a multi-channel, multi-link, multi-level and highly complex regulatory process, in which many signaling pathways, such as AR signaling pathway, STAT3 signaling pathway, Wnt/β-catenin signaling pathway, play diverse roles in the process of EMT mediated PCa metastasis (Fearon et al., 2012; Murata and Kang, 2018; Shorning et al., 2020). Recent studies have found that lncRNAs in PCa could participate in the regulation of key genes involved in the above EMT-related signaling pathways. In order to clarify the regulatory network between them, this review summarized the research progress of EMT-related signaling pathways regulated by lncRNAs in PCa in recent years.

## AR Pathway

Androgen and AR are two main pathogenic factors of PCa. AR is a member of the nuclear receptor superfamily, which mainly exists in the nucleus and belongs to steroid receptors. Androgen is the main ligand of AR. When the ligand is lacking, AR is mainly distributed in the cytoplasm, binding to heat shock proteins and sustaining an inactive state (Husmann et al., 1990; Sar et al., 1990; Heery et al., 1997). When androgen enters the cell, AR will dissociate from heat shock protein, releasing ligand binding domain and spontaneously dimerizing. Then dimerized AR enters the nucleus and bind to ARE (Androgen Response Elements) on target genes to exert its transcription regulatory role.

The interaction network between AR and lncRNAs is multi-dimensional instead of unidirectional. AR is a transcription factor and there existed a positive feedback loop between AR and AR-induced lncRNAs in PCa cells. Zhang *et al.* (Zhang J et al., 2018) identified ARLNC1 (AR-regulated long non-coding RNA 1) as a target lncRNA of AR. They demonstrated that AR induced the expression of ARLNC1 and ARLNC1 stabilized AR via direct RNA-RNA interaction, and silencing ARLNC1 could reduce the global activity of AR signaling and the viability of PCa cells. Huntingtin-interacting protein 1 (HIP1) is an important AR regulator that could modulate the transcriptional activity of AR (Mills et al., 2005). Two research groups have reported similar positive feedback loops, mediated by HIP1, between AR and AR-induced lncRNAs. Shi *et al.* (Shi et al., 2021) and Li *et al.* (Mather et al., 2021) both treated LNCaP cells and VCap cells with 10 nM dihydrotestosterone, identifying both PCLN16 and PCAL7 could suppress the migration and proliferation of PCa cells. Functionally, they found PCLN16 and PCAL7 were transcriptionally induced by AR, and both the two lncRNAs could interact with HIP1 and reduce HIP1 degradation, advancing AR signaling via positive feedback loops.

Another important regulatory mechanism of the expression of AR protein is via ubiquitination or de-ubiquitination. The ubiquitination process of AR is mainly mediated by the E3 ubiquitin ligase MDM2, a well-known regulator of TP53. Previous studies reported that LncRNA LINC00675 and HOTAIR were markedly upregulated in androgen-insensitive PCa cell lines and CRPC patients, blocking the interaction of AR proteins with MDM2 and thereby preventing AR ubiquitination and protein degradation (Zhang et al., 2015; Yao et al., 2020). LncRNA PCBP1-AS1 (Zhang B et al., 2021) was reported to be able to stabilize AR via promoting the stability of USP22-AR complex by binding to AR NTD domain and enhancing AR de-ubiquitination. Importantly, targeting LINC00675, HOTAIR and PCBP1-AS1 all could effectively inhibit the growth and invasion ability of PCa cells and restore the drug sensitivity of Enzalutamide-resistant PCa cells, which offered distinct view for future clinical utilization.

Besides increasing the stability of AR protein, lncRNAs were also reported to affect the stability of AR mRNA and subsequent translation. Lnc-OPHN1-5, located closely to AR on chromosome X, was an AR-suppressor and could elevate Enzalutamide (Enz) sensitivity via inhibiting AR signaling (Zhang M et al., 2021). The study demonstrated that lnc-OPHN1-5 competed with hnRNPA1 to bind to the 3′UTR site of AR mRNA, not only decreasing the half-life period of AR mRNA but also blocking the enrichment of AR mRNA in the ribosome RNA 18S. PVT1 is a widely reported lncRNA in PCa which is over-expressed and related to an unwilling prognosis (Yang et al., 2017; Liu et al., 2021). Videria *et al.* (Videira et al., 2021) found that PVT1 may participate in an AR-dependent transcriptional repression program in PCa cells. They demonstrated that 160 genes repressed by AR were up-regulated and the epigenetic markers such as H3K27me3 and H3K27ac were remodeled when PVT1 was silenced. Therefore, they speculated that PVT1 may assist AR in repressing the expression of tumor suppressor genes via altering the epigenetic mode in PCa cells.

Taken together, the interaction network between AR and lncRNAs is multi-directional. AR can induce the transcription of certain lncRNAs, and lncRNAs could also regulate the expression abundance of AR at mRNA or protein level. Such reciprocal mode inside the AR-lncRNAs network may indicate us more potential methods to improve current anti-androgen therapies.

## STAT3 Signaling Pathway

STAT3 is a member of the signal transducer and activator of transcription (STAT) family. STAT family has dual functions of signal transduction and transcriptional activation. In JAK-STAT signal pathway, JAK (Janus kinase) and STATs are intracellular and receptor binding proteins, which complete signal transduction from cytoplasm to nucleus (Darnell et al., 1994). Generally, STAT3 signaling pathway is activated by extracellular stimulus, especially diverse cytokines, including IL-6, IL-8, CXCL-5, and COX2, from the surrounding tumor micro-environment (Lou et al., 2000; Tong et al., 2017; Roca et al., 2018; Zheng P et al., 2018). While non-canonical regulation of STAT3 pathway always occurred inside cells involving the phosphorylation of Y705 site or S727 site and the ubiquitination of STAT3 (Bae et al., 2016; Wang et al., 2018). The continuously activated STAT3 signaling pathway plays an important role in tumorigenesis, including human PCa (Mora et al., 2002; Kroon et al., 2013; Schroeder et al., 2014). As reported by Nicholas *et al.*(Don-Doncow et al., 2017), 95% of 223 metastatic PCa samples were positive staining of p-STAT3 and the bone metastases presented an extremely obvious expression of p-STAT3 compared with lymph nodes or visceral metastases samples, suggesting the crucial role of active STAT3 in promoting the metastasis of PCa.

MAGI2-AS3 was a significantly reduced lncRNA in PCa samples, and it acted as the miR-424-5p sponge to suppress the progression of PCa by regulating COP1 expression and STAT3 signaling pathway activation (Wei et al., 2022). Jiang *et al.* (Jiang et al., 2021) reported that LINC00467 can promote PCa cell growth and metastasis via two methods. Firstly, silencing LINC00467 in tumor-associated lymphocytes (TAMs) promoted TAM polarization to the M2 subtype and inhibited the metastatic capacity of co-cultured PCa cells. Secondly, LINC00467 could promote PCa cell growth and metastasis via sponging miR-494-3p and subsequently activating the expression of STAT3. Zhang *et al.* found lncAMPC promoted the metastasis via a dual function in the cytoplasm and nucleus. They proved that cytoplastic lncAMPC could induce the expression of LIF by binding and suppressing the activity of miR637. While lncAMPC in the nucleus could remove histone H1.2 from the distal promoter region of LIFR and therefore up-regulated LIFR level. The axis of LIF/LIFR was a well-known inducer of JAK1-STAT3 signal (Nicola and Babon, 2015; Chan et al., 2019). Due to the up-regulated expression of LIF/LIFR axis under the dual stimulation of lncAMPC, JAK1-STAT3 signaling pathway was activated with the ensuing promoted expression of metastasis-associated genes, ultimately leading to the metastasis of PCa (Zhang et al., 2020). Zhu *et al.* also reported the lncRNA-activator role of STAT3 in PCa. STAT3 induced the expression of LINC00160, then LINC00160 bound to EZH2, leading to the hypermethylation of RCAN1, and therefore promoted the proliferation and metastasis of PCa cells (Zhu et al., 2022).

In conclusion, besides the canonical ceRNA interaction network of lncRNA-miRNA-mRNA, there also may exist a reciprocal interaction between lncRNAs and STAT3. As a transcription activator, STAT3 could induce the expression of certain lncRNAs, such as LINC00160, to epigenetically regulate the expression mode of down-stream genes. Besides, some lncRNAs could indirectly activate STAT3 pathway via regulate the expression of proteins up-stream of STAT3 signaling pathway, such as lncAMPC.

## Wnt/β-Catenin Signaling Pathway

Wnt gene is the combined term of homologous genes Int and Wingless. The Wnt protein encoded and expressed by Wnt gene is a collection of secretory glycoprotein families including 19 Wnt protein members (Ring et al., 2014). Wnt signaling pathway is composed of transcription regulatory factors, functional proteins and enzymes, and *β*-Catenin is a key component of Wnt signaling pathway (Watanabe et al., 2004). *β*-Catenin is a multifunctional effector protein and its N-terminal and C-terminal have binding sites with glycogen synthase kinase-3 (GSK-3) and T-cell factor/lymphoid enhancing factor (TCF/LEF), respectively. It plays an important role in maintaining cell adhesion and the morphological structure of adjacent tissues. (Mao et al., 2014).

Canonical Wnt/β-Catenin pathway plays an important role in different stages of tumor development, including cancer cell proliferation, migration, invasion, tumorigenesis and metastasis. Normally, the cytoplastic *β*-Catenin complex consists of Axin, APC and CK1α and GSK-3β. The complex is encapsulated with E3 ubiquitin ligase *β*-TRCP and eventually degraded by the ubiquitin-proteome pathway (Jang et al., 2015). Wnt1 ligands (Wnt2, Wnt3, Wnt3a and wnt8a) bind to FZD receptor and LRP5/6 to initiate Wnt signaling pathway. Subsequently, the scattered proteins in the cytoplasm are phosphorylated and form a complex with Axin, which binds to GSK3β to block its activation (Liu et al., 2015). This combination further results in the breakdown of the degradation complex, leading to the accumulation of *β*-catenin in the cytoplasm. The accumulated *β*-catenin is transported to the nucleus, which is recognized as the main event of canonical Wnt pathway activation, and then interacts with TCF/LEF to form an activator complex, thereby initiating the transcription of Wnt/β-catenin signal transduction target genes, including c-Myc, Cyclin D1 and MMP-7 (Yoshida and Saya, 2014).

Noncoding RNA activated by DNA damage (NORAD) is a recently identified lncRNA that could promote PCa metastasis via regulating Wnt/β-catenin (Zhang Y et al., 2018). The ceRNA interaction of NORAD and miR-30a-5p promoted the growth and metastasis, while silencing the expression of NORAD exerted the opposite effect. AGAP2-AS1 (AGAP2 antisense RNA 1) is another lncRNA that was reported to activate Wnt/β-catenin pathway. Zhao *et al.* (Zhao et al., 2021) reported that AGAP2-AS1 could form a feedback loop with miR-628-5p/FOXP2 and promote proliferation and EMT progression of PCa by activating the Wnt signaling pathway.

Moreover, besides the canonical Wnt/β-Catenin pathway, the non-canonical Wnt pathway also participates in the EMT progression of PCa. As a non-canonical Wnt signaling pathway ligand, Wnt5a works with its receptor FZD2 and induces the expression of EMT-related genes in PCa. LncRNA MCM3AP-AS1 has been demonstrated abnormally up-regulated in PCa tissues and could up-regulate the expression of Wnt5a at mRNA and protein level via sponging miR-876-5p in PCa cells (Wu G et al., 2020).

## PTEN/PI3K/AKT Signaling Pathway

The deficiency of PTEN (phosphatase and tensin homolog on chromosome 10) tumor suppressor and the oncogenic activation of PI3K (phosphatidylinositol-4,5-bisphosphate 3-kinase) signaling axis are among the most common altered signaling pathways in primary PCa that facilitate tumor occurrence, disease progression and therapeutic resistance (Shorning et al., 2020). Recent studies showed that nearly 15–20% PCa patients presented a loss of function of PTEN and the rate may reach 40–60% when the disease progress into the CRPC or metastatic stage (Yoshimoto et al., 2012; Leinonen et al., 2013; Jamaspishvili et al., 2018). PI3K is mainly composed of the regulatory subunit p85 and the catalytic subunit P110a and could specifically catalyze phosphatidylinositol. The difference between catalytic subunit P110 and related substrates can be divided into class I, II, and III subtypes. The study of type I PI3K showed that activated PI3K targeted PIP2 (phosphatidylinositol diphosphate), located on the plasma membrane, and then transformed into PIP3. Then PIP3, as the second messenger, binds to the downstream protein kinase B (AKT) containing PH domain and PDK1, prompting PDK1 to phosphorylate the Ser308 site of AKT and then activate AKT (Huang et al., 2014). Phosphorylated AKT can activate NF-κB, CREB and other transcription factors to activate anti-apoptotic genes to inhibit cell apoptosis and play a positive role in cell proliferation. Therefore, Akt is regarded as an important hub factor in PI3K/AKT signaling pathway. In human PCa, the phosphorylation of AKT at Ser473 has been reported to be an excellent indicator of the prognosis of PCa patients (Ayala et al., 2004) and the interaction of IGF-I/PI3K/AKT signaling pathway and AR signaling pathway promote the synthesis of PSA (Liu et al., 2011). Moreover, Lamin A/C protein could regulate the activation and inactivation of PTEN to promote the migration and invasion of PCa cells (Yan and Huang, 2019). Taken together, The dysregulated PTEN/PI3K/AKT signaling pathway can regulate the synthesis of various proteins and is involved in the proliferation and apoptosis, migration and differentiation of PCa cells (Wu et al., 2018).

LncRNA MBNL1-AS1 exerted a positive regulation of PTEN. As reported, MBNL1-AS1 is down-regulated in PCa cells, and the MBNL1-AS1/miR-181-5p/PTEN axis could suppress the proliferation and migration ability of PCa cells through the inhibitory effect of PTEN on AKT phosphorylation (Ding et al., 2021). LncRNA-ATB is a stimulator of EMT associated with ZEB1 and ZNF217 expression and regulates the EMT progression of PCa *via* activation of ERK and PI3K/AKT signaling pathways (Xu et al., 2016). In addition, Wu *et al.* (Wu et al., 2017) analyzed the differentially expressed lncRNAs in three pairs of PCa specimens and found that LINC01296, a novel identified highly expressed in PCa, was related to preoperative PSA (*p* = 0.002), lymph-node metastasis (*p* = 0.035), Gleason score (*p* = 0.001), tumor stage (*p* = 0.036) and recurrence-free survival of PCa patients. *In vitro* biological experiments have further proved that knockdown of LINC01296 can inhibit the proliferation, migration and invasion of PCa cells by inhibiting the activity of the PI3K/AKT/mTOR pathway. Another study (Sun et al., 2021) revealed that lncRNA DANCR exerted an oncogenic role in PCa cells via regulating the miR-185-5p/LASP1 axis and activated the PI3K/AKT/GSK3β pathway. Activated GSK3β promoted the transcriptional activity of Snail, leading to the elevated migration and invasion ability of PCa cells.

## TGF-β/Smad Signaling Pathway

The TGF-β signaling pathway is a transmembrane signal transduction pathway widely existing in invertebrates and vertebrates. TGF-β belongs to a polypeptide superfamily with extensive and complex biological activity regulation. It plays an important role in the regulation of body tissue homeostasis and early embryonic development. The abnormal activation of TGF-β superfamily signaling pathway is related to a variety of diseases, including human cancers, fibrotic diseases, autoimmune diseases, etc. (Shangguan et al., 2012; Xue et al., 2014; Fennen et al., 2016). The Smad family is an important type of transcriptional regulator, and Smad2 and Smad3 are the key downstream factors of TGF-β. Activated TGF-β binds to TGF-β receptor on the cell membrane, catalyzes the phosphorylation and activation of Smad2 and Smad3, and then Smad2/3 and Smad4 bind to the nucleus and regulate target gene transcription (Park et al., 2015; Zhao et al., 2020). In the early stage of tumor, TGF-β signal can suppress tumor growth by inhibiting the secretion of cytokines and growth factors, and inducing apoptosis. When the tumor progresses to the pathological stage, due to the loss or mutation of heterozygous alleles, the inhibitory effect of TGF-β signal disappears, and it plays a role in promoting cancer (Oh and Joo, 2020). Specifically, TGF-β up-regulates the expression levels of VEGF and bFGF, and simultaneously induces the expression of MMP-2 and MMP-9. MMPs are enzymes that could promote the degradation of the vascular endothelial basement membrane and promote the neovascularization of solid tumors, which greatly benefits tumor distant metastasis. Studies have reported that the expression of TGF-β1 and Smad2 are higher in PCa tissues than that in prostate epithelial tissues. The high expression of TGF-β1 can induce cell metastasis, invasion and cause adverse effects on the overall survival of PCa patients (Atılgan et al., 2016).

LncRNA ANRIL was overexpressed in PCa, and knockdown of ANRIL significantly decreased the levels of TGF-β1 and p-Smad2 and inhibited the proliferation and migration of PCa cells (Zhao et al., 2018). The low expression of lncRNA DGCR5 acted as a predictor of poor survival in PCa. While over-expressing DGCR5 could decrease the stemness of PCa cells with the down-regulation of TGF-β1, and supplementing TGF-β1 could reverse the effect of over-expressing DGCR5 (Li B et al., 2019). Zhang *et al.* (Zhang H et al., 2019) reported that lncRNA MIR4435-2HG could promote the migration and invasion of PCa cells and the treatment of TGF-β inhibitor attenuated the enhancing effects of over-expression MIR4435-2HG in PCa cells. Distant bone metastasis is a well-recognized lethal event of advanced PCa patients. PCAT7, a bone metastasis-related lncRNA, could activate TGF-β/Smad signaling via elevating the protein level of TGFBR1 with sponging miR-324-5p and the activated TGF-β/Smad signaling in turn could induce the expression of PCAT7 by promoting the formation of a Smad3/Smad4/SP1 complex, which demonstrated a positive feedback loop in promoting the bone metastasis in PCa cells (Lang et al., 2020).

## NF-κB Signaling Pathway

NF-κB signaling pathway is a widely reported pathway mainly involved in immune or inflammatory regulation. Canonical NF-κB signaling pathway consists of the several sequential events:the activation of IKK complex (containing catalytic subunits IKKα and IKKβ and regulatory subunit NEMO), phosphorylation of IκBα (activated by IKK complex), degradation of IκBα protein and release of NF-κB and the nuclear shuttle of NF-κB. The aberrant activation of NF-κB signaling pathway is also reported to participate in the alteration of diverse tumor phenotypes (Wang et al., 1996; Chen et al., 1999; Yamamoto and Gaynor, 2001; Karin and Greten, 2005). In human PCa, lncRNA DRAIC is reported to inhibit the progression of PCa via suppressing the activation of NF-κB (Saha et al., 2020). As reported, DRAIC obstruct the formation of IKK complex via directly interact with IKKα subunit and NEMO subunit, alleviating the phosphorylation of IκBα protein and the nuclear transportation of NF-κB. The inactivation of NF-κB signaling pathway by DRAIC could efficiently inhibit the growth and metastasis ability of human PCa cells. Moreover, Shang *et al.* (Shang et al., 2019) reported lncRNA PCAT1 could activate the NF-κB signaling pathway in CRPC cells. They firstly identified PCAT1 as an up-regulated and prognosis-related lncRNA in CRPC samples and found PCAT1 could compete with PHLPP to bind to the C-terminal tetratricopeptide repeat (TPR) domains of FKBP51 protein in an androgen-deprive environment. This competitive perturbation of FKBP51 and PHLPP not only abolished the suppression of AKT by PHLPP but also strengthened the stability of FKBP51/IKKα complex, resulting in the constant activation of NF-κB signaling pathway and finally the progression of PCa disease.

## Other Signaling Pathways or Effective Proteins

The above is the main lncRNAs-regulated signaling pathways that were mostly reported to participate in the EMT process of human PCa cells, while researches about other signaling pathways, especially the pathways targeting EMT process, are not so abundant. Here, we selected the three most typical ones to make a brief discussion. The first one is NORAD. In addition to regulating Wnt/β-catenin signaling pathway, lncRNA NORAD also could regulate the release of extracellular vesicles (EVs) to promote the bone metastasis of PCa cells (Hu W et al., 2021). Via regulating miR-541-3p-PKM2 axis, NORAD could affect the release of EVs through phosphorylating SNAP-23 and promote the internalization of EVs by up-regulating the production of ATP in EVs. Elevated EVs containing PKM2 finally induced the bone metastasis of PCa cells. Secondly, NEAT1-1 functioned as a bridge to connect CYCLIN1 and CDK19 via a m6A dependent manner (Wen et al., 2020). The special complex promoted the phosphorylation of Pol II Ser2 and transcription of RUNX2 to induce distant metastasis of PCa cells. The third one is LINC00261, which perform a dual-role both in cytoplasm and in nucleus like lncAMPC (Mather et al., 2021). In the cytoplasm, LINC00261 promoted the expression of CBX2 via sponging miR-8485 through a ceRNA network. While in the nucleus, LINC00261 acted as a scaffold to induce SMAD-driven expression of the FOXA2. The hyper-activation axis of LINC00261-CBX2-FOXA2 coordinatively promoted the proliferation and metastasis of PCa cells. Other pathways or key factors that could interact with certain lncRNAs in PCa still deserve more interest for further exploration.

## Discussion

**FIGURE 1:**
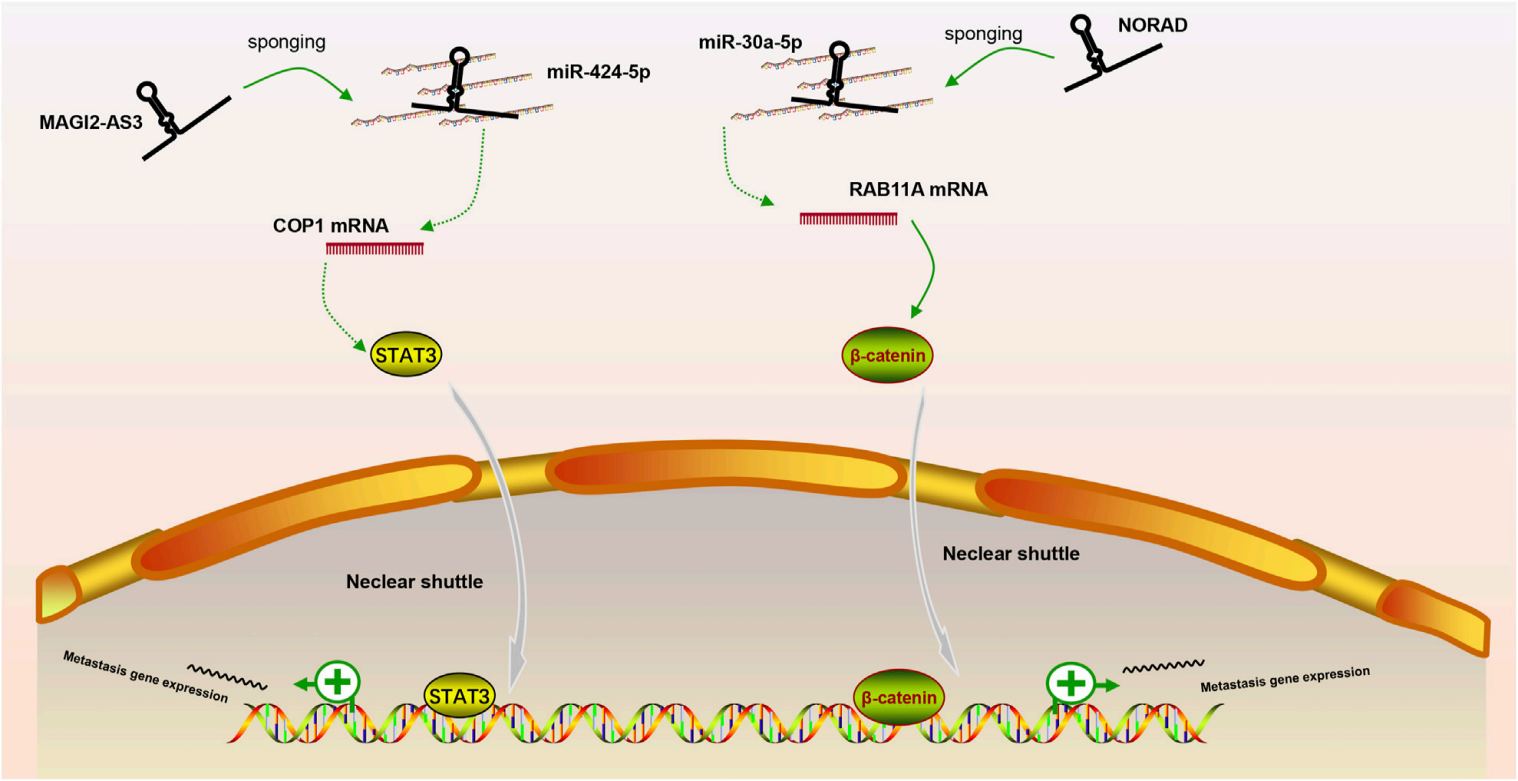
The ceRNA network of lncRNA in regulating downstream signaling pathway. MAGI2-AS3 and NORAD regulate STAT3 or Wnt/β-catenin signaling pathway via sponging miR-424-5p or miR-30a-5p, respectively.

**FIGURE 2:**
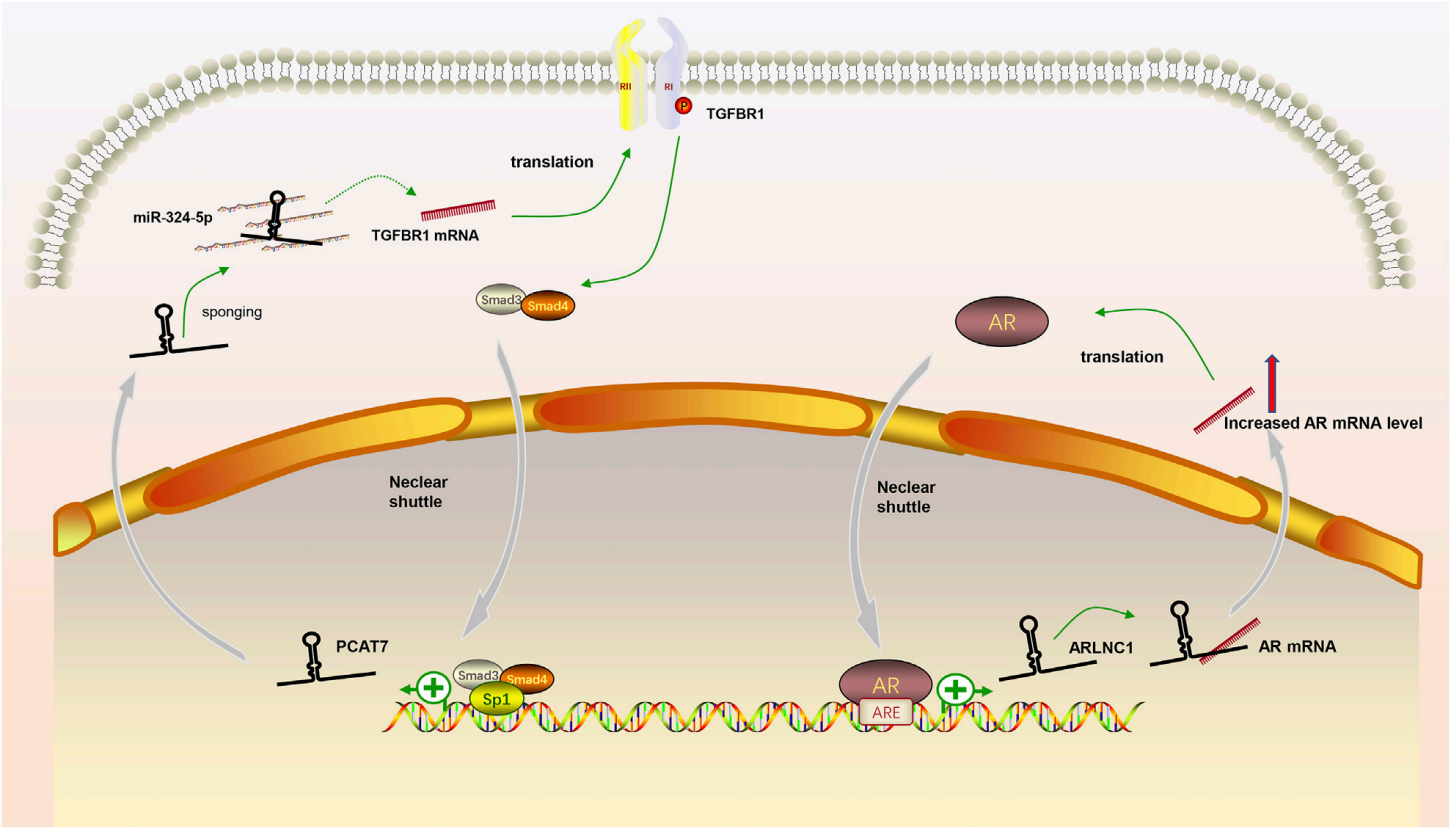
The positive feedback loop between lncRNAs and key signaling effectors. (1) AR induced the expression of lncRNA ARLNC1 and ARLNC1 increased the stability of AR mRNA to promote AR signaling pathway; (2) LncRNA PCAT7 increased the level of TGFBR1 and activated TGF-β/Smad signaling pathway promoted the expression of PCAT7 via the formation of Smad3/Smad4/SP1 complex.

**FIGURE 3:**
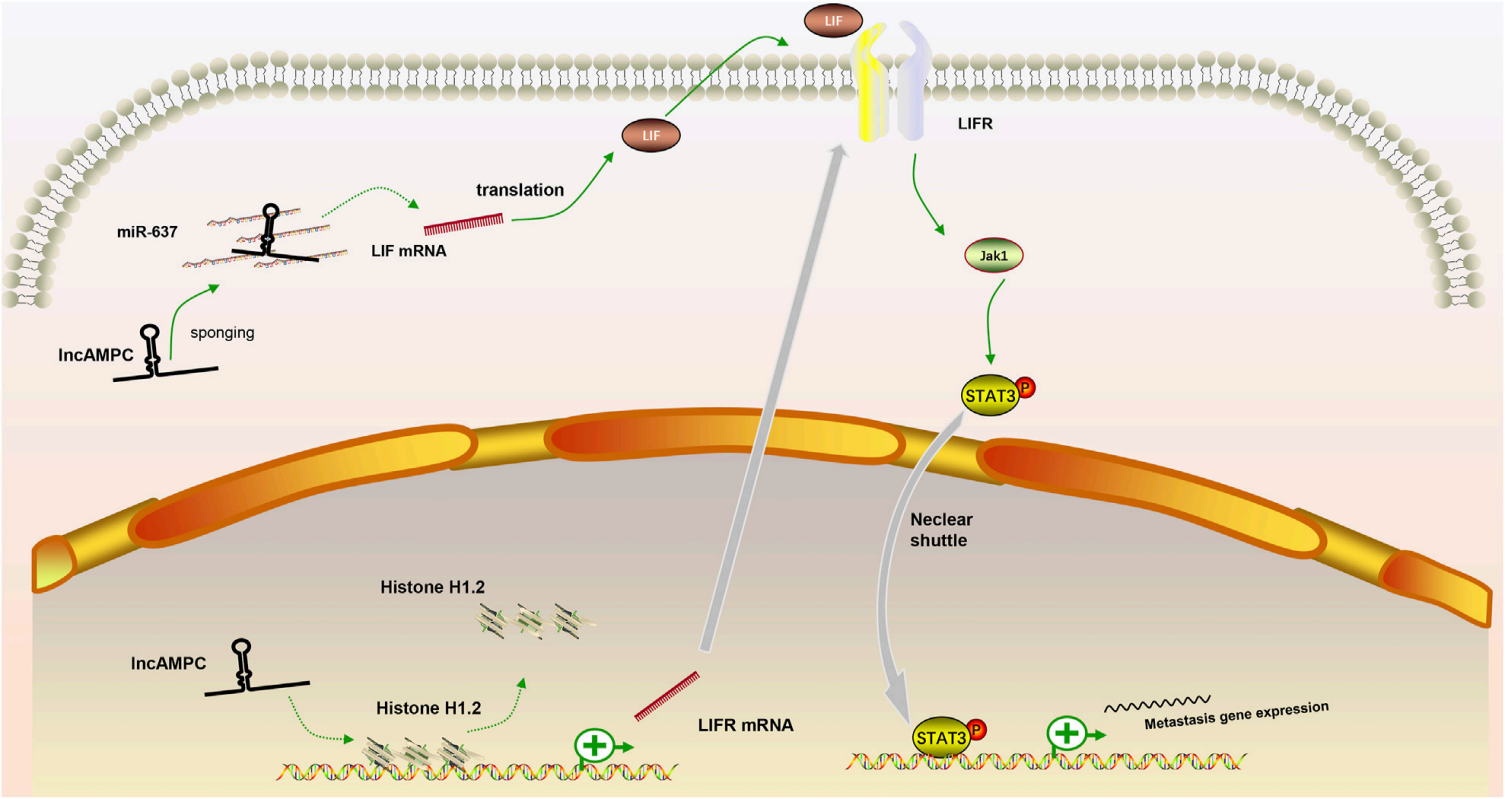
The positive feedback loop between lncRNAs and key signaling effectors. (1) In the cytoplasm, lncAMPC upregulated LIF expression by sponging miR-637; (2) In the nucleus, lncAMPC enhanced LIFR transcription by decoying histone H1.2 away from the upstream sequence of the LIFR gene; (3) The increased binding of LIF and LIFR stimulated the JAK1-STAT3 pathway to promote metastasis-associated gene expression.

**FIGURE 4:**
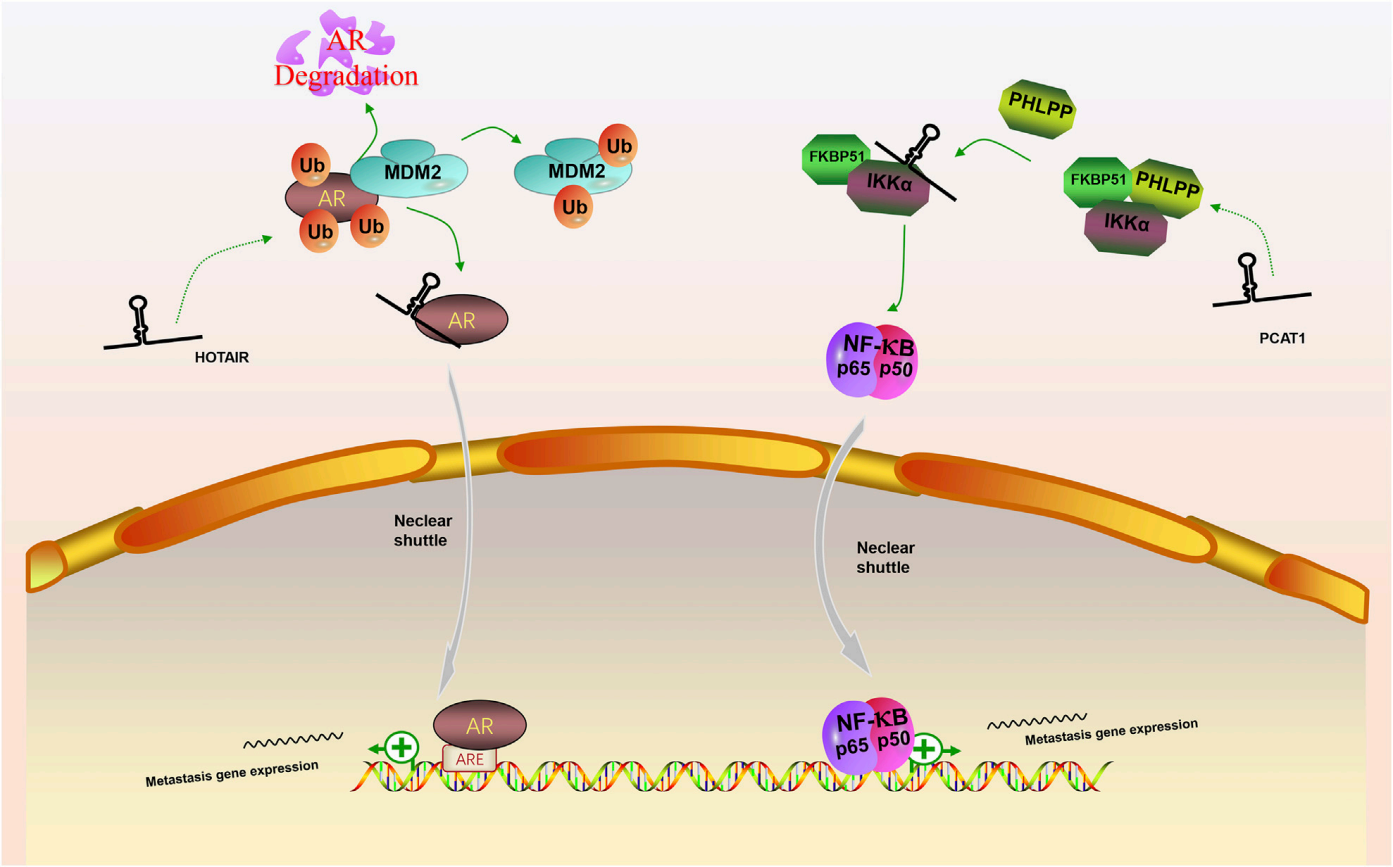
The competing integration of the binding site between lncRNAs with specific regulators to promote the stability of key signaling effectors. (1) LncRNA HOTAIR interfered the degradation of AR by MDM2 and promote the AR signaling pathway; (2) LncRNA PCAT1 activate NF-κB signaling pathway via abolishing the inhibitory effect of PHLPP on IKKα.

In our review, we discuss the role of lncRNAs in participating in the EMT process in PCa via regulating multiple signaling pathways, including AR signaling pathway, STAT3 signaling pathway, Wnt/β-catenin signaling pathway, PTEN/PI3K/AKT signaling pathway, TGF-β/Smad pathway and NF-κB signaling pathway. Based on our discussion, there are four modes of how lncRNAs participate in the EMT process of PCa via regulating diverse signaling pathways: 1) Forming a ceRNA network via sponging miRNAs to alleviate the repression of downstream target genes and therefore activate or inactivate related signaling pathways, such as MAGI2-AS3 in regulating STAT3 signaling pathway and NORAD in regulating Wnt/β-catenin signaling pathway (shown in Figure 1); 2) Forming a positive feedback loop with key signaling effectors, such as the interaction between ARLNC1 with AR and PCAT7 with TGF-β/Smad signaling pathway (shown in Figure 2); 3) Epigenetically regulating the transcription of down-stream genes, for example, lncAMPC in regulating STAT3 signaling pathway (shown in Figure 3) or PVT1 in regulating AR signaling pathway; 4) Competing the binding site with regulator to promote the stability of key signaling effectors, namely HOTAIR in blocking the degradation of AR and PCAT1 in activating of NF-κB (shown in Figure 4).

Moreover, considering that metastasis is the main lethal cause of PCa patients, we hope clinical doctors and researchers could benefit from our current literature review. From our standpoint, among the signaling pathways we discussed above, lncRNAs targeting AR signaling pathway are of much more values and here we raised several unsolved points that deserve further exploration. 1) Bioinformatic analysis or clinical researches about lncRNAs that could predict the metastasis-free survival (MFS) is absent. As distant metastasis is universally regarded as the terminal status of PCa patients, researches that are lack of quantitative analysis of MFS generally failed to evaluate the progress of PCa patients, since only MFS has been shown to be a surrogate endpoint for overall survival (OS) (Xie et al., 2020; Gharzai et al., 2021). 2) Researches about lncRNAs that targeting distant metastasis is deficient. The lncRNAs reported above are mainly differentially expressed in samples of public database, which neglecting the particularity of metastatic samples. 3) Researches about lncRNAs that could promote adjuvant therapies are scarce. Latest research demonstrated that radiotherapy combined with ADT help to improve MFS without compromising quality of life (Kishan et al., 2022). However, we find that researches focusing on the role of lncRNAs in promoting radiotherapy in PCa are very sparse. Therefore, we think these may be new directions for future researches.
